# Supplementary material for: YFR016c/Aip5 is part of an actin nucleation complex in yeast
Source: Biol Open. 2019 Jul 30;8(8):bio044024. doi: 10.1242/bio.044024 (PMC6737964; doi:10.1242/bio.044024)
Supplement: Supplementary information [file biolopen-8-044024-s1.pdf]

Biology Open (2019): doi:10.1242/bio.044024: Supplementary information

Figure S1

A

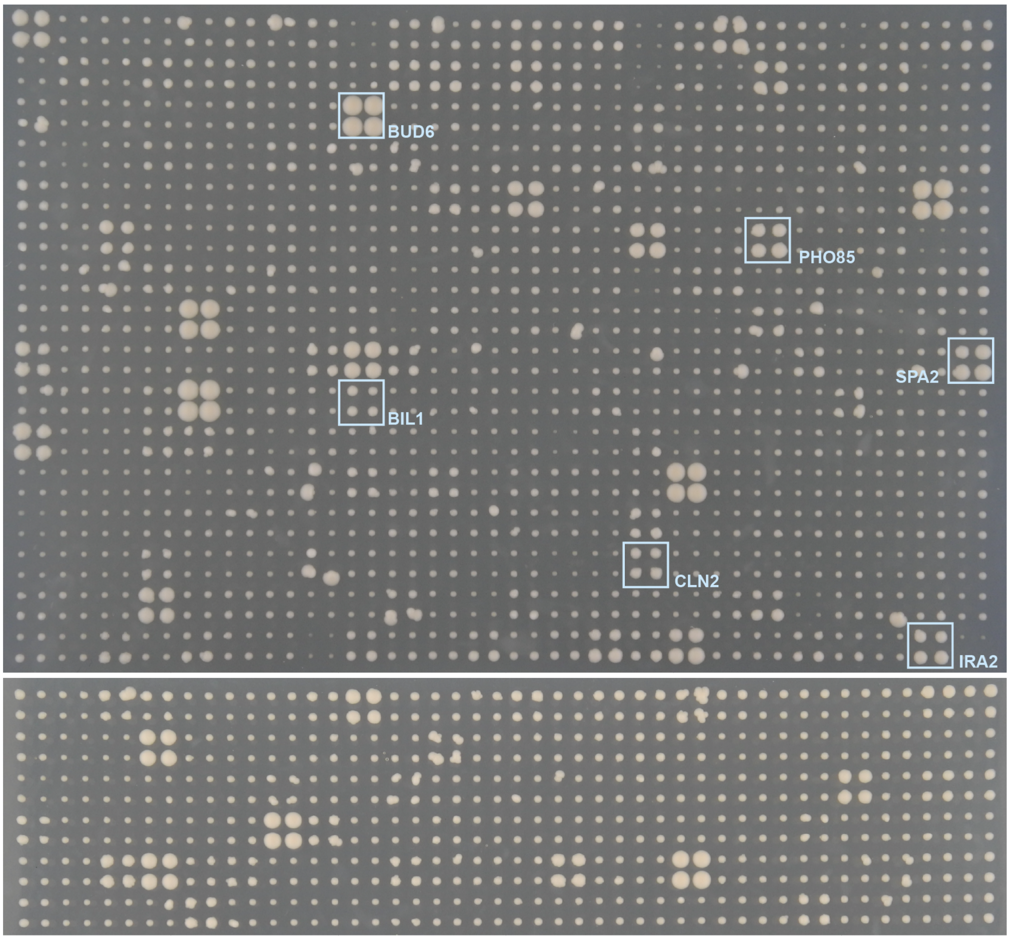

B

ABP1	KEL1	ASK10	LDB17	BFA1	MEC1	BNI4	MSS4	BUD5	NIS1	CDC28	PFY1	CLA4	PSY4	CRZ1	RHO5	EDE1	SAC6	FKS1	SEC2	HCM1	SHS1	INP52	SMI1
SSD1	BNI5	STT4	SRO7	TEF1	CHS3	UBP7	BNI1 STOP	YBR238C	VMA13	NAS2	CIK1	CAP1	SCC2	CDC25	PLC1	PEX4(P)	CLB1	YPD1	SPC24	CYR1	BUB3	KSS1	LCB4
AFR1	KEL2	BBC1	LRE1	BIK1	MKK1	BNR1	MYO1	BUD6	NUM1	CDC3	PIK1	CLB2	PTC1	CYK3	RIM21	ELM1	SAC7	FKS2	SEC3	HLR1	SIT4	IPL1	SMP1
SSK1	SVL3	SWE1	SHE4	TOR2	PPH3	VAC8	IQG1 STOP	YGR058W	CYK3 ΔPPP	ZDS1	SPC72	BUD8	SEC10	SMT3(P)	PDS1	GUK1(P)	CLB3	FUS2	SPC110	SEC15	CSE4	NHA1	LCB5
AIP1	KIC1	BCK1	LRG1	BIM1	MLC2	BOI1	MYO2	BUD9	ORC6	CDC37	PIL1	CMD1	RAD9	DBF2	RLM1	END3	SAK1	FUS3	SFH1	HOF1	SKG6	IPP1	SMY1
SSK2	AVO2(P)	SWI4	TOR1	TOS2	SHO1(P)	VHS2	AXL2 STOP	YKL161C	ASE1	ZDS2	ARP6	BCY1	KAR3	SEC62(P)	PNC1	TLG2(P)	APC2	PHO85	DUO1	TPK1	SPC19	PTC2	AUR1
AMN1	KIN1	BCK2	LSP1	BIR1	MOB1	BUB2	MYO5	CBK1	PAC1	CDC5	PKC1	CMK1	RAX1	DBF20	ROM2	ENT2	SCP1	GIC1	SFH2	HOG1	SKN7	IQG1	SOG2
STE11	TCO89	SWI6	APP1	TPM1	BOI1PH (P)	VRP1	SHO1 STOP	YMR124W	CIN1	BOI2	SMC1	APG17	DYN1	CDC24	ELG1	UBC6(P)	MPS1	NBP2	DAD1	GPB2	SPR28	IRA1	TSC13
ARC1	KIN2	BEM1	LTE1	BIT61	MSB1	BUD14	NAP1	CDC10	PAM1	CDC6	PKH1	CMP2	RGA2	DOP1	RSR1	FAB1	SCP160	GIN4	SFH3	HSL1	SKT5	IRS4	SPA2
STE20	AVO1	SYP1	NAS2	TPM2	BOI1SH3 (P)	YBR053C	HOF1 ΔFCH	YOR304C	KIP3	SLA1	MCD1	ACF2	BUD20	Actin wt(P)	CLB4	SIZ1	STU2	MSN4	SLI15	SEC4	CDC20	RAS2	ELO1
ARC40	KIN4	BEM2	MAD2	BMH1	MSB3	BUD2	NBA1	CDC11	PAN1	CHS1	PKH2	CNA1	RHO1	DSE1	RVS161	FAR1	SDP1	GLG1	SFH4	HSL7	SLA2	IST2	SPH1
SVL3	ROM1	TAO3	LST8	TSC11	INN1	YDL173W	WSC1 STOP	YPK1	PAC10	STE50	IRR1	MLC1(P)	GPA2	SSO1	CLN1	CPR6	DAM1	MSN2	HSK3	PDE1	CHK1	RAS1	LAG1
Aktin mut(P)	KIP2	BEM3	MAD3	BMH2	MSB4	BUD3	NET1	CDC12	PBS2	CHS2	PKH3	CNB1	RHO2	DUN1	RVS167	FKH1	SEC1	GSY1	SFH5	HYM1	SLM1	KAR9	SPT5
STE5	HUA1	TCB1	KOG1	TUS1	BNR1 STOP	YDL203C	MID2 STOP	ZEO1	GIM4	SRO77	ESP1	TEM1(P)	IME2	SNC1(P)	CLN2	PUF3	STU1	HOT1	DAD2	TOK1	TEL1	GPB1	LAC1
ARP1	LAS17	BEM4	MCK1	BNI1	NBA1	BUD4	NIP1	CDC14	PEA2	CIN8	PRK1	CRN1	RHO4	ECM25	SAC1	FKH2	SEC14	GUS1	SFK1	INP51	SLT2	KCC4	SRV2
STE7	YEL023C	TDH1	LEER(P)	UBC9	CYK3 ΔSH3	YDR348C	PFK1	AIR1	VHS1	EXO84	SCC4	CDC42(P)	RGS2	PEX19(P)	CLN3	HSP82	SPC34	MSN1	DAD3	SKO1	SKM1	IRA2	

PAC11	SMC3	CRM1	NUF2	DPL1	PHO80	ATS1	ELP4	TUM1	UBA4	NCS2	NCS6	URM1	BSP1	IKI1	ELP2	ELP3	ELP6	YLR 031W	MSO1	NUP57	NUP85	SEH1	NSP1
NIC96	NUP49	NUP145	GSP1	SCS2	VAC17	MMR1	RGD1	SIR2	EXO70	SEC5	SEC6	SEC7	SEC8	SEC9	SEC13	SEC17	GLC7	YPT11	YPT31	YPT32	SHE2	SHE3	INP2
VPS1	MID2	WSC1	COY1	CSE1	NEW1	SFB3	NOP4	MRC1	RLP7	PPZ2	YAK1	GIC2	AIM44	TCB2	TCB3	SIC1	OSH1	OSH2	OSH3	OSH4	OSH5	OSH6	OSH7
CHC1	LEM3	DNF1	DNF2	RGA1	GPH1	EGD1	CLC1	MYO3	MYO4	MYO2 STOP	MYO3 STOP	MYO4 STOP	MYO5 STOP	MDM10	ULS1	Pnativ BOI1	Pnativ BOI2	Pnativ BEM1	Pnativ BEM3	Pnativ NBA1	Pnativ STE20	Pnativ CLA4	Pnativ NIS1
Pnativ HOF1	NAT1	SSB1	SSB2	SSZ1	ZUO1	RPL26A	BTT1	PXL1	GLC8	SDS22	YFR 016C	RCY1	MAP1	MAP2	RPL17B	EGD2	SRP54	STE4	MON2	RGD2	RGL1	GDI1	SEC27
RER1	DSL1	DSL3	SCS22	BET3	BET5	YPT1 CEN	BEM3 Δ140	BOI1 WK	BOI1 PL	BOI2 WK	BOI2 PL	NBA1 255-	NBA1 100-	NBA1 AXXP	SEC3 AXXP	RGA2 AXXP	TRS20	TRS23	TRS31	TRS65	TRS85	TRS120	TRS130

***In vivo* interaction screen of Aip5.** Upper panel: Split-Ubiquitin analysis of 533 diploid Aip5 CRU-containing yeast strains each co-expressing a different N_{ub} fusion protein under a copper-inducible promoter. The N_{ub} fusion proteins are enriched for proteins functioning in cell polarity, exocytosis, stress response and the actin cytoskeleton. The cells were mated, spotted in quadruplets and finally transferred onto medium containing 5-FOA and 150 µM copper to induce the expression of the N_{ub} fusions. Cells were incubated for 4 days. Positive hits are indicated in light blue and are: Bud6, Spa2, Cln2, Bil1, Pho85, Ira2. False positives and chaperones are removed from the list of interactors (Hruby et al, 2011). Lower panel: Positions and identities of N_{ub}-fusions on the array.

Figure S2

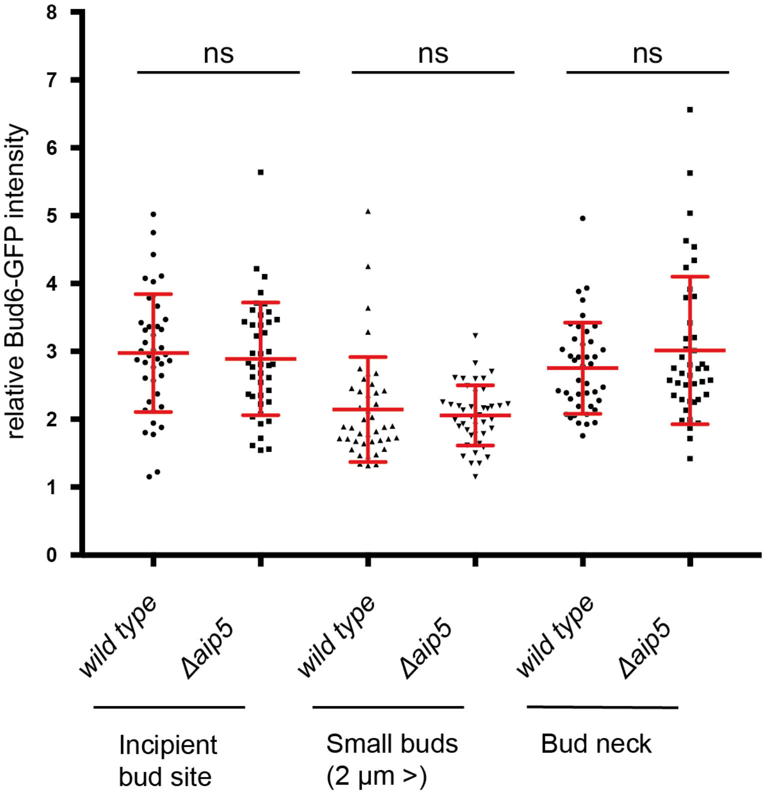

Aip5 does not influence the localization of Bud6. Time-resolved images of Bud6-GFP in wildtype (n=42)- or *Δaip5*-cells (n=42) were acquired to calculate the relative intensity at the presumptive bud site, at the tips of small buds (<2µm) and at the bud neck. Images of 5 z-stacks were taken every 3 minutes over several cell cycles with a wide-field microscope and stacked to a sum projection for the analysis. Relative fluorescence intensity was normalized to the cytosol of the mother cell. Statistical analysis derived from two independent measurements was performed with a Kruskal-Wallis test followed by a Dunns post test. Mean values and standard deviation are shown in red.

Figure S3

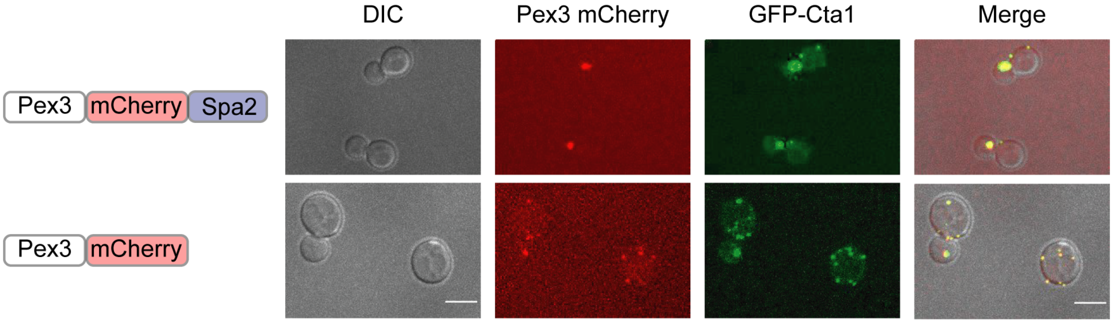

Pex3-mCherry-Spa2 localizes to peroxisomes. Colocalization of Pex$_{1-45}$mCherry-Spa2 with the peroxisomal marker GFP Cta1. Confocal fluorescence microscopy images were taken in 10 z stacks and stacked to maximum projections. Scale bar indicates 5 µm. Note that Spa2-decorated peroxisomes form large cluster at random sites in the cytosol. Scale bar indicates 5µM.

Biology Open • Supplementary information

Table S1. Yeast and *E.coli* strains.

Strain	Genotype	Source	Identifier
E.coli XL1 blue	endA1 gyrA96(NalR) thi-1 recA1 relA1 lac glnV44 F' [::Tn10 proAB+ lacIq (lacZ)M15] hsdR17(rK- mK+)	Stratagene	XL1 blue
E.coli Bl21 DE3	F- ompT gal dcm lon hsdSB(rB-mB-) λ(DE3) pLysS(CmR)	Invitrogen	BL21 DE3
Yeast JD47	*MATa, his3-Δ200, leu2-3, 112 lys2-801, trp1-Δ63, ura3-52*	(Dohmen et al., 1995)	JD47
Yeast JD53	*MATα, his3-Δ200, leu2-3, 112 lys2-801, trp1-Δ63, ura3-52*	(Dohmen et al., 1995)	JD53
JD47 AIP5 CRU	AIP5::AIP5-CRU, HIS3	This study	STY218
JD53 N_{ub}-Bud6, *Δspa2*	BUD6::pCUP1-N_{ub}-HA-BUD6, kanMX4, SPA2::CmLEU2	This study	YAD617
JD53 N_{ub}-Kel1, *Δspa2*	KEL1::pCUP1-N_{ub}-HA-KEL1, kanMX4, SPA2::CmLEU2	(Neller et al., 2015)	YAD616
JD53 N_{ub}-empty, *Δspa2*	pCUP1-*Nub-HA kanMX4, CEN,* SPA2::CmLeu2	(Neller et al., 2015)	YAD618
JD53 N_{ub}-Spa2, *Δbud6*	SPA2::pCUP1-N_{ub}-HA-SPA2, kanMX4, BUD6::CmLEU2	This study	YAD614
JD53 N_{ub}-Kel1, *Δbud6*	KEL1::pCUP1-N_{ub}-HA-KEL1, kanMX4, BUD6::CmLEU2	This study	YAD613
JD53 N_{ub}-empty, *Δbud6*	pCUP1-*Nub-HA kanMX4, CEN,* BUD6::CmLEU2	This study	YAD615
JD47 AIP5 GFP, SPA2 mCherry	AIP5::AIP5 GFP, TRP1, SPA2::SPA2 mCherry, URA3	This study	YOG95
JD47 AIP5 GFP, *Δspa2*	AIP5::AIP5 GFP, TRP1, SPA2::hphNT1	This study	YOG63
JD47 AIP5 GFP, *Δbud6*	AIP5::AIP5 GFP, TRP1, BUD6::natNT2	This study	YOG424
JD47 Pex3$_{1-45}$mCherrySpa2, *Δspa2*	SPA2::hphNT1, ura3::URA3, PMET17-Pex3$_{1-45}$-mCherry-Spa2	This study	YOG324
JD47 Pex3$_{1-45}$mCherry, *Δspa2*	SPA2::hphNT1, ura3::URA3, PMET17-Pex3$_{1-45}$-mCherry	This study	YOG369
JD47 *Δbud6*	BUD6::natNT2	This study	ARY191
JD47 *Δaip5 (hph)*	AIP5::hphNT1	This study	YOG59
JD47 *Δaip5(nat),* Bud6 GFP	BUD6::BUD6GFP, TRP1, AIP5::natNT2	This study	YOG153

JD47 *Δaip5Δbud6*	AIP5::hphNT1, BUD6::natNT2	This study	YOG265
JD47 *Δtpm1*	TPM1::hphNT2	This study	YAD1340
JD47 *Δaip5Δtpm1*	TPM1::hphNT2, AIP5::natNT2	This study	YOG475
JD47 AIP5 GFP	AIP5::AIP5GFP, TRP1	This study	YOG69
JD47 $AIP5_{\Delta 1000\text{-}1125}$	$AIP5_{\Delta 1000\text{-}1125}$	This study	YAD3444
JD47 $AIP5_{\Delta 1000\text{-}1125}$ *Δtpm1*	$AIP5_{\Delta 1000\text{-}1125,}$ TPM1::hphNT1	This study	YAD3447
JD47 $AIP5_{\Delta 1000\text{-}1125}$ CRU	$AIP5_{\Delta 1000\text{-}1125}$:: $AIP5_{\Delta 1000\text{-}1125}$ CRU, HIS3	This study	YOG533

Biology Open • Supplementary information

Table S2. Plasmid list.

Plasmid	Description	Source
pMet17-Cub-R-Ura3-pRS313 empty	pRS 313, lacZ, AmpR, His3	(Hruby et al., 2011, Sikorski, Hieter, 1989)
pAC	pT7-HIS6, kanMXR	(Iffland et al., 2000)
pGex6P1	pTAC-GST, ampR	Amersham
pFA hphNT1	pAgTEF-*hphNT1, Amp*^{R}	(Janke et al., 2004)
pFA natNT2	pAgTEF-*natNT2*, *Amp*^{R}	(Janke et al., 2004)
pFA CmLEU2	pAgTEF-*CmLEU2, Amp*^{R}	(Bahler et al., 1998)
N_{ub} empty cen	pCUP1-*Nub-HA kanMX4, CEN, Amp*^{R}	(Hruby et al., 2011)
pMet17-GFP(S65T)-pRS313 empty	pRS313, lacZ, AmpR, His3	(Tian, Wu & Johnsson, 2014)
GST Bud6$_{1-141}$	GST-Bud6$_{1-141}$ pGex-2T	This study
GST Bud6$_{1-364}$	GST-Bud6$_{1-364}$ 6P1	This study
His$_{6}$ AIP5$_{1000\text{-end}}$	His$_{6}$ –TEV site- AIP5$_{1000\text{-end}}$ pAC	This study
Cub-R-Ura3-pRS 303 empty	pRS 303, lacZ, AmpR, His3	(Wittke et al., 1999)
AIP5 Cub-R-Ura3-pRS303	AIP5 Cub-R-Ura3 pRS303	This study
pMet17-Aip5$_{1-1233}$-Cub-R-Ura3 pRS313	pMet17-Aip5$_{1-1233}$-Cub-R-Ura3 pRS313	This study
pMet17-Aip5$_{1-300}$-Cub-R-Ura3 pRS313	pMet17-Aip5$_{1-300}$-Cub-R-Ura3 pRS313	This study
pMet17-Aip5$_{1-600}$-Cub-R-Ura3 pRS313	pMet17-Aip5$_{1-600}$-Cub-R-Ura3 pRS313	This study
pMet17-Aip5$_{1-1000}$-Cub-R-Ura3 pRS313	pMet17-Aip5$_{1-1000}$-Cub-R-Ura3 pRS313	This study
pMet17-Aip5$_{300-1233}$-Cub-R-Ura3 pRS313	pMet17-Aip5$_{300-1233}$-Cub-R-Ura3 pRS313	This study
pMet17-Aip5$_{600-1233}$-Cub-R-Ura3 pRS313	pMet17-Aip5$_{600-1233}$-Cub-R-Ura3 pRS313	This study
pMet17-Aip5$_{1000-1233}$-Cub-R-Ura3 pRS313	pMet17-Aip5$_{1000-1233}$-Cub-R-Ura3 pRS313	This study
pMet17-Aip5$_{1125-1233}$-Cub-R-Ura3 pRS313	pMet17-Aip5$_{1125-1233}$-Cub-R-Ura3 pRS313	This study
pMet17-Aip5$_{1000-1131}$-Cub-R-Ura3 pRS313	pMet17-Aip5$_{1000-1131}$-Cub-R-Ura3 pRS313	This study
pMet17-Aip5$_{1-1233}$ GFP(S65T) pRS313	pMet17-Aip5$_{1-1233}$ GFP(S65T) pRS313	This study
pMet17-Aip5$_{1-300}$ GFP(S65T) pRS313	pMet17-Aip5$_{1-300}$ GFP(S65T) pRS313	This study
pMet17-Aip5$_{1-600}$ GFP(S65T) pRS313	pMet17-Aip5$_{1-600}$ GFP(S65T) pRS313	This study

Biology Open • Supplementary information

pMet17-Aip5$_{1-1000}$ GFP(S65T) pRS313	pMet17-Aip5$_{1-1000}$ GFP(S65T) pRS313	This study
pMet17-Aip5$_{300-1233}$ GFP(S65T) pRS313	pMet17-Aip5$_{300-1233}$ GFP(S65T) pRS313	This study
pMet17-Aip5$_{600-1233}$ GFP(S65T) pRS313	pMet17-Aip5$_{600-1233}$ GFP(S65T) pRS313	This study
pMet17-Aip5$_{1000-1233}$ GFP(S65T) pRS313	pMet17-Aip5$_{1000-1233}$ GFP(S65T) pRS313	This study
pMet17-Pex3$_{1-45}$-mcherry-mcs, pRS306	pMet17-Pex3$_{1-45}$-mCherry-mcs, pRS306, Ura3, AmpR	This study, idea based on (Luo, Zhang & Guo, 2014)
pMet17-Pex3$_{1-45}$-mcherry-Spa2, pRS306	pMet17-Pex3$_{1-45}$-mCherry-Spa2, pRS306, Ura3, AmpR	This study
Aip5 GFP, pRS304	Aip5 GFP, pRS304, Trp1, AmpR	This study
Bud6 GFP, pRS304	Bud6 GFP, pRS304, Trp1, AmpR	Tis study
Spa2 mCherry, pRS306	Spa2 mCherry, pRS306, Ura3, AmpR	(Neller et al., 2015)
N$_{ub}$ Bud6$_{1-364}$ cen	Pcup N$_{ub}$ HA kanMX Bud6 $_{1-364}$, Cen	This study
N$_{ub}$ Bud6 $_{360-end}$ cen	Pcup N$_{ub}$ HA kanMX Bud6 $_{360-788}$, Cen	This study
N$_{ub}$ Bud6$_{1-240}$ cen	Pcup N$_{ub}$ HA kanMX Bud6 $_{1-240}$, Cen	This study
N$_{ub}$ Bud6$_{1-141}$ cen	Pcup N$_{ub}$ HA kanMX Bud6 $_{1-141}$, Cen	This study
pML104	backbone: pRSII426	(Laughery et al., 2015)
pML107	*Cen*, Amp^{R}, LEU2	(Laughery et al., 2015)
pML107 Bem1-561	pML107	This study
pML107 Boi1-304	pML107	This study
P$_{CTA1}$- GFP Cta1, pRS 314	pRS314	This study

Table S3. Primer list.

Primer	Sequence 5´->3
YFR016c Cub Eag	CCTCCCGGCCGCTCTACTCCAATCACTAG
YFR016c Cub SAL	CCACCGTCGACCCAATAGTATCGTAAATGAGTTCC
YFR016c Cub Ctr 450	CGA ATG AGT TTT TAG AGC AAC
Cub ctr	TCTTCTAGCTGCTTACCG
G1 YFR016c	GTTTCTGAACACGAAATTGTCG
G2 YFR016c	CTATAAATCACATGTGCTGTC
S1 YFR016c	GCAATTATTGTACTGGTGTGCTTGATTACTCTGGATTAAATAAAGAGTGACATG cgtacgctgcaggtcgac
S2 YFR016c	CACATGTGCTGTCAAGAGATTATGTTAGTTACGTAGCGCGCTTAAGCTTTCTTA atcgatgaattcgagctcg
YFR016c_EagI_atg_fw	ccttCGGCCGCCATGGTAGAATCTTTGACTGTAG
YFR016c_Glu301at	cctccCGGCCGCCATGGAGATCTTAAATGTTAATGA

Biology Open • Supplementary information

g_EagI_fw	GGG
YFR016c_Glu600atg_EagI_fw	cctccCGGCCGCCATGGAAGATCAAGAAGGGGAAC
YFR016c_Lys1000atg_EagI_fw	ccttCGGCCGCCATGAAGGAATTGGAAGTAGAAGAAAC
YFR016c_Lys1125atg_ EagI_fw	cctccCGGCCGCCATGAAGCCACAAGACGTAATAACG
YFR016c_Asn300_SalI_rv	CCACCGTCGACCCATTCTCTTCTCGAGGTAAGG
YFR016c_Glu600_SalI_rv	CCACCGTCGACCCTTCTTTGGCAACCTCGAGATC
YFR016c_Lys1000_SalI_rv	CCACCGTCGACCCCTTGTCTTCTCCTTTATTATCC
YFR016c_1132 Salrv	CCACCgtcgacCCCGTTATTACGTCTTGTGGCTTG
YFR016c_Lys1000_SfiI_TEV_fw	CctccGGCCAGCACGGCCGAAAACCTGTACTTCCAGGGTAAGGAATTGGAAGTAGAAGAAAC
YFR016c_end_Sfi1_rv	cctccGGCCAAAAAGGCCGCTTTCTTAAATAGTATCGTAAATG
S1 Bud6	GCTGGCTGCCAAATTGGTGTAGTAATCCTCGTATTATTTTAAAATTAGATGcgtacgctgcaggtcgac
S2 Bud6	AAGCCAAAAGCACTAATCTCTTTTCCGTTAGCTTTCATAAAATTAGTGTATTTAatcgatgaattcgagctcg
G1 Bud6	TCG CTA GCC GAG CTG AAC GAA T
G2 Bud6	CTG ACA GCT TAT GAT GCT GAT AAG G
S1 Spa2	ACGAGCCACCGAAACAGAATAAACAAAAGAAAAGAAAGAGTAAACcgtacgctgcaggtcgac
S2 Spa2	ATTGTCTTTGTCTTCCTTTTCTTTCTCCTCTAGATACTACTAACTatcgatgaattcgagctcg
G1 Spa2	CTGCGCCGGGTGATGCGTGACG
G2 Spa2	CCA AGG ATA GTG GGT GTT CTC
H3	GGAAGGAGTTAGACAACCTG
N3	CTCAGGTATAGCATGAGGTCGC
AgTefp RV	catgtcgctggccgggtgac
GFP (S65T)ATG+84RV	CGC CTT CAC CCT CTC CAC TGA C
pMet fw	TTTCCTTCGTGTAATACAGGG
Bud6 ATG FW SalI	cgatGTCGACCATGAAGATGGCCGTGGAT
Bud6 141 RV Acc65I	cgat GGTACC TTACCGATGCTCTTGCTGCAT
Bud6 240 RV Acc65I	cgatGGTACCTTATGAAAGTGCATCATCATCATC
Bud6 364 Acc65I Rv	ggaggggtaccTTATACTGGACAAGTTCCATTC
Bud6aip3 Nuisal1fw	CCACCCGTCGACCACTTGTCCAGTATTTTTA
Bud6 end RV Acc65	GACGGggtaccTTAAGTAAACCCCGGCCCAAAAT
Pex3_atg_EagI_fw	CctccCGGCCGATGGCCCCAAATCAAAGATC
Pex3_Lys45_XbaI_rv	cctccTCTAGATTTATACAACCATCTCTTCACG
Spa2 GST fw1 BamHI	CCACCGGATCCATGGGTACGTCAAGCGAG

Biology Open • Supplementary information

Spa2_HA_SalI_rv	CCTCCgtcgacCCagcgtaatctggaacatcgtatgggtaCTTCAACTTCGAATTCAAATAATTTATTTC
gR1_YFR3309	GATCAGTCTTTCGCATCTAGTGATGTTTTAGAGCTAG
gR2_YFR3309	CTAGCTCTAAAACATCACTAGATGCGAAAGACT
doYFRd1000-1125	CCAGAATTGGAAAAACAAGATATAAAGGATAATAAAGGAGAAGACAAGCCACAAGACGTAATAACGACTAGCGAGATCCGGAAGCTGAAT
YFR016cCRISPctrl2860	CTAAGATTGCTGAGGCGCTC

Biology Open • Supplementary information

References

Bahler, J., Wu, J.Q., Longtine, M.S., Shah, N.G., McKenzie, A.,3rd, Steever, A.B., Wach, A., Philippsen, P. & Pringle, J.R. 1998, "Heterologous modules for efficient and versatile PCR-based gene targeting in Schizosaccharomyces pombe", *Yeast (Chichester, England),* vol. 14, no. 10, pp. 943-951.

Dohmen, R.J., Stappen, R., McGrath, J.P., Forrova, H., Kolarov, J., Goffeau, A. & Varshavsky, A. 1995, "An essential yeast gene encoding a homolog of ubiquitin-activating enzyme", *The Journal of biological chemistry,* vol. 270, no. 30, pp. 18099-18109.

Hruby, A., Zapatka, M., Heucke, S., Rieger, L., Wu, Y., Nussbaumer, U., Timmermann, S., Dunkler, A. & Johnsson, N. 2011, "A constraint network of interactions: protein-protein interaction analysis of the yeast type II phosphatase Ptc1p and its adaptor protein Nbp2p", *Journal of cell science,* vol. 124, no. Pt 1, pp. 35-46.

Iffland, A., Tafelmeyer, P., Saudan, C. & Johnsson, K. 2000, "Directed molecular evolution of cytochrome c peroxidase", *Biochemistry,* vol. 39, no. 35, pp. 10790-10798.

Janke, C., Magiera, M.M., Rathfelder, N., Taxis, C., Reber, S., Maekawa, H., Moreno-Borchart, A., Doenges, G., Schwob, E., Schiebel, E. & Knop, M. 2004, "A versatile toolbox for PCR-based tagging of yeast genes: new fluorescent proteins, more markers and promoter substitution cassettes", *Yeast (Chichester, England),* vol. 21, no. 11, pp. 947-962.

Laughery, M.F., Hunter, T., Brown, A., Hoopes, J., Ostbye, T., Shumaker, T. & Wyrick, J.J. 2015, "New vectors for simple and streamlined CRISPR-Cas9 genome editing in Saccharomyces cerevisiae", *Yeast (Chichester, England),* vol. 32, no. 12, pp. 711-720.

Luo, G., Zhang, J. & Guo, W. 2014, "The role of Sec3p in secretory vesicle targeting and exocyst complex assembly", *Molecular biology of the cell,* vol. 25, no. 23, pp. 3813-3822.

Neller, J., Dunkler, A., Rosler, R. & Johnsson, N. 2015, "A protein complex containing Epo1p anchors the cortical endoplasmic reticulum to the yeast bud tip", *The Journal of cell biology,* vol. 208, no. 1, pp. 71-87.

Sikorski, R.S. & Hieter, P. 1989, "A system of shuttle vectors and yeast host strains designed for efficient manipulation of DNA in Saccharomyces cerevisiae", *Genetics,* vol. 122, no. 1, pp. 19-27.

Tian, C., Wu, Y. & Johnsson, N. 2014, "Stepwise and cooperative assembly of a cytokinetic core complex in Saccharomyces cerevisiae", *Journal of cell science,* vol. 127, no. Pt 16, pp. 3614-3624.

Wittke, S., Lewke, N., Muller, S. & Johnsson, N. 1999, "Probing the molecular environment of membrane proteins in vivo", *Molecular biology of the cell,* vol. 10, no. 8, pp. 2519-2530.
